# Comparison of the ERP-Based BCI Performance Among Chromatic (RGB) Semitransparent Face Patterns

**DOI:** 10.3389/fnins.2020.00054

**Published:** 2020-01-31

**Authors:** Shurui Li, Jing Jin, Ian Daly, Cili Zuo, Xingyu Wang, Andrzej Cichocki

**Affiliations:** ^1^Key Laboratory of Advanced Control and Optimization for Chemical Processes, Ministry of Education, East China University of Science and Technology, Shanghai, China; ^2^Brain-Computer Interfacing and Neural Engineering Laboratory, School of Computer Science and Electronic Engineering, University of Essex, Colchester, United Kingdom; ^3^Skolkowo Institute of Science and Technology, Moscow, Russia; ^4^Systems Research Institute, Polish Academy of Sciences, Warsaw, Poland; ^5^Department of Informatics, Nicolaus Copernicus University, Toruń, Poland

**Keywords:** brain-computer interface, ERP, chromatic stimuli, semitransparent face, visual stimuli

## Abstract

**Objective:**

Previous studies have shown that combing with color properties may be used as part of the display presented to BCI users in order to improve performance. Build on this, we explored the effects of combinations of face stimuli with three primary colors (RGB) on BCI performance which is assessed by classification accuracy and information transfer rate (ITR). Furthermore, we analyzed the waveforms of three patterns.

**Methods:**

We compared three patterns in which semitransparent face is overlaid three primary colors as stimuli: red semitransparent face (RSF), green semitransparent face (GSF), and blue semitransparent face (BSF). Bayesian linear discriminant analysis (BLDA) was used to construct the individual classifier model. In addition, a Repeated-measures ANOVA (RM-ANOVA) and Bonferroni correction were chosen for statistical analysis.

**Results:**

The results indicated that the RSF pattern achieved the highest online averaged accuracy with 93.89%, followed by the GSF pattern with 87.78%, while the lowest performance was caused by the BSF pattern with an accuracy of 81.39%. Furthermore, significant differences in classification accuracy and ITR were found between RSF and GSF (*p* < 0.05) and between RSF and BSF patterns (*p* < 0.05).

**Conclusion:**

The semitransparent faces colored red (RSF) pattern yielded the best performance of the three patterns. The proposed patterns based on ERP-BCI system have a clinically significant impact by increasing communication speed and accuracy of the P300-speller for patients with severe motor impairment.

## Introduction

Brain-computer interface (BCI) systems enable their users to achieve direct communication with others or the outside environment by brain activity alone, independent of muscle control. There are many potential user groups for BCI systems, including, but not limited to, individuals living with amyotrophic lateral sclerosis (ALS) who are in the locked-in state (LIS).

The brain activity used to control a BCI can be measured using different signal acquisition approaches such as electroencephalogram (EEG), magnetoencephalography (MEG), functional magnetic resonance imaging (fMRI), electrocorticogram (ECoG), or near infrared spectroscopy (NIRS) (Vidal, 1973, 1977; Wolpaw et al., 2002). Since EEG signals are recorded via non-invasive electrodes placed on the surface of the scalp, EEG-based BCI systems are very commonly used. Three key signal components of the EEG are frequently used for BCI control: event-related potentials (ERPs), steady-state visual evoked potentials (SSVEP), and motor imagery (MI) (Sutton et al., 1965; Coles and Rugg, 1995). The focus of the present study is the ERP-based BCI.

The P300 speller, is a visual ERP-based BCI system, that can elicit a P300 ERP component using an Oddball paradigm. The P300 potential is the largest positive deflection with a latency around 300 ms after the oddball stimulus onset, and is associated with various cognitive processes such as attention, working memory, and executive function (Van Dinteren et al., 2014). In addition, P300-based BCI systems can evoke P100, N200, and N400 components. The P300 speller was originally described by Farwell and Donchin (1988). In this study, participants were requested to watch a screen displaying a 6 × 6 matrix containing 26 letters and 10 digits. They were asked to focus on the rare target stimuli and ignore the common non-target stimuli. Stimuli were flashed (highlighted) in a row-column pattern (RCP). However, the RCP results in the adjacency-distraction and double-flash problems, which can cause false positive P300 ERPs during flashes of non-target stimuli that are adjacent to the target. Thus, some researchers investigated ways to avoid this issue, and strengthen the performance of the P300 BCI system.

For example, Takano et al. identified that the color of the stimuli could influence P300-speller system performance. They replaced the white/gray flicker matrix with a green/blue flicker matrix and found that the chromatic stimulus improved the performance of the P300-speller system (Takano et al., 2009). Jin et al. (2012) proposed a set of stimuli patterns that made use of images of the face with different emotional content and degrees of movement, including neutral faces, smiling faces, shaking neutral faces, and shaking smiling faces. The results revealed that BCIs that make use of face-based stimuli paradigms are superior to the traditional RCP. Kaufmann et al. (2011) attempted to overlay characters used in a P300 speller with semitransparent images of familiar faces. This resulted in a higher classification accuracy by evoking N170 and N400 ERPs. The N170 is a negative voltage deflection occurring approximately 200 ms after stimulus onset, which is generally related to motion of the stimuli (Jin et al., 2015), speech processing (Niznikiewicz and Squires, 1996), and vocabulary selection (Kutas and Hillyard, 1980). The N400 component occurs at 300-500 ms post-stimulus, and is connected with face recognition (Kaufmann et al., 2011) and language understanding (Johnson and Hamm, 2000). The influences produced by stimuli have also been reflected in other factors, such as, but not limited to, the inter-stimulus intervals (Sellers et al., 2006), stimulus intensity (Cass and Polich, 1997), and stimulus motion (Sutton et al., 1965; Martens et al., 2009). A large number of works have attempted to design optimal paradigms based on face stimuli to improve the performance of BCI systems. For example, Li et al. (2015) observed that compared with a paradigm that only used semitransparent famous faces, the green semitransparent famous face paradigm could lead to improved classification performance. Based on this, we further explore the performance differences between red semitransparent face (RSF), green semitransparent face (GSF), and blue semitransparent face (BSF) patterns. In addition, Guo et al. (2019) investigated how red, green, and blue (RGB) colors may be used as stimuli in a new layout of flash patterns based on single character presentation. They reported that the red stimuli paradigm yielded the best performance. Thus, we hypothesize that faces, that are colored red, can produce a higher classification accuracy compared to patterns that combine red, green, and blue colors with faces.

Although a large number of works have attempted to design optimal paradigms to improve the performance of BCI systems, there are scarce studies on the pattern of chromatic difference and face combination. In our new patterns, the flashing row or column in the BCI display grid is overlaid with semitransparent faces that are colored red, green, or blue and we compare the effect of these three new spelling patterns on BCI performance. In addition, we investigate the ERP waveforms induced by the proposed “red semitransparent face” (RSF), “green semitransparent face” (GSF), and “blue semitransparent face” (BSF) patterns and evaluate the classification performance among the three patterns.

## Materials and Methods

### Participants

Twelve healthy participants (S1–S12, five females and seven males, aged 22–25 years, mean 24 years old) with normal or corrected to normal vision volunteered for the current study. All participants’ native language is Mandarin Chinese, and they are familiar with the Western characters used in the display. They are all right-handed and had normal color vision. Before the experiment began, all participants provided informed consent via a process which the local ethics committee approved. Two participants’ data was abandoned because the accuracy of three patterns were all lower than 60%. According to Kubler et al. (2004), these two participants may be described as “BCI-illiterate.” Four of the ten participants (S1, S3, S6, and S7) had participated in a BCI experiment previously. All participants were informed of the whole experimental process in advance.

### Experimental Design

**FIGURE 1:**
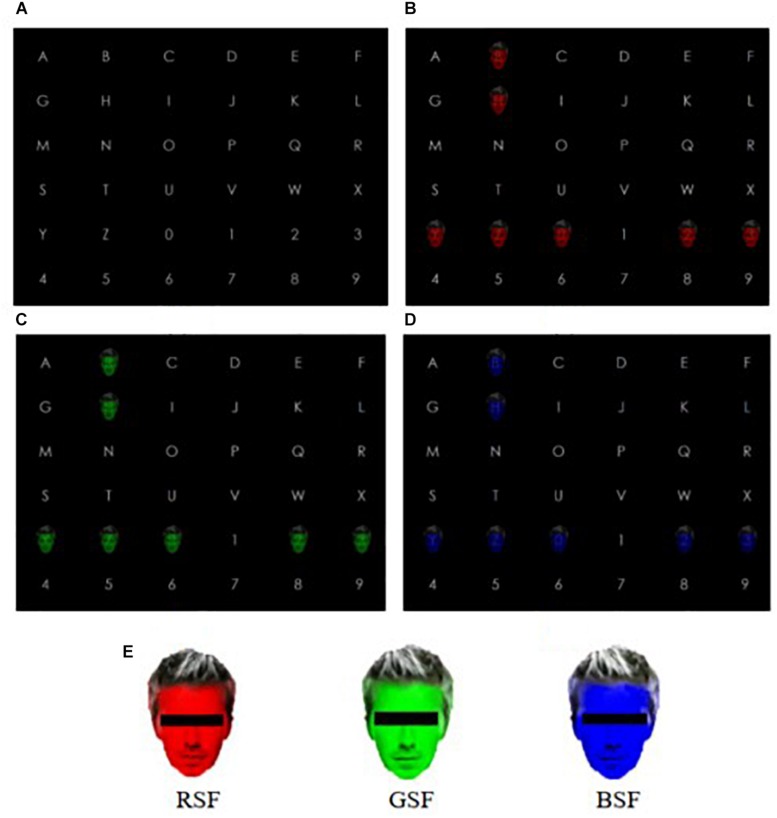
The experimental pattern. **(A)** Character matrix; **(B)** Red semitransparent face (RSF) pattern; **(C)** Green semitransparent face (GSF) pattern; **(D)** Blue semitransparent face (BSF) pattern; **(E)** the legend of the three stimuli. Note that in order to avoid copyright infringement, faces are portrayed with censor boxes. (During the experiment censor boxes were not presented). In addition, **(B–D)** presented the fifth flash.

A 20-inch LCD monitor (Lenovo LS2023WC) with standard RGB gamut and 1600 × 900 resolution was used for stimuli presentation. Its maximum luminance was set to 200cd/m^2^. In the experiment, we instructed participants sit approximately 105 cm in front of the display, which was 30 cm tall (visual angle: 16.3°) and 48 cm wide (visual angle: 25.7°) in a quiet laboratory which was relatively dim with the optic intensity of the environment approximately 40lx. Participants were asked to relax themselves and avoid unnecessary movement throughout the experiment. The graphical interface of the BCI was developed using “Qt Designer 4.8” software. The semitransparent images of faces, painted with three primary colors, red (255,0,0), green (0,255,0), and blue (0,0,255), were selected as stimuli, as shown in the Figure 1, and the transparency was set to 50%. The stimulus onset asynchrony (SOA) was set to 250 ms, and the stimulus interval was set to 100 ms throughout all stages of the experiment.

Figure 1A shows the interface of the 6 × 6 spelling matrix before the experiment began; it contains 26 letters and 10 digits. The parameters of the three patterns including background color, the appearance and distance of characters and the stimuli style remain the same throughout the experiment. In Figure 1B, the pattern showed a semitransparent face colored red as the stimulus covered the characters. For the sake of convenience, we refer to this as the RSF pattern. Figure 1C shows the semitransparent face colored green as the stimulus covered the characters. This is referred to as the GSF pattern. Figure 1D shows the semitransparent face colored blue as the stimulus covered the characters. This is called the BSF pattern. In addition, Figures 1B–D presented the fifth flash.

In the current study, three patterns were presented to participants in sequence. During the experiment, participants were requested to silently count the number of times target characters flashed. The stimulus presentation pattern is based on binomial coefficients (Jin et al., 2010, 2014a). The formulation is *C*(*n*,*k*) = *n*!/*k*!(*n*−*k*)!, 0≤*k*≤*n*, where *n* refers to the number of flashes per trial and *k* refers to the number of flashes per trial for an element in the matrix. In this study, the combination of *C*(12,2) was used to represent the 12-flash pattern. Table 1 describes the coding of the stimulus sequence in the 12-flash pattern with 36 flash pattern pairs. The locations in Table 1 correspond to the locations of the 36 characters in Figure 1A. Specifically, the first pair (1,4) in Table 1 means the first and the fourth flash will cover character “A”. During the offline and online block – for each of the three patterns – the presentation sequences for each stimulus are consistent with Table 1.

**TABLE 1:** The coding of stimulus sequence of the 12-flash pattern.

(1,4)	(1,5)	(1,6)	(1,7)	(1,8)	(1,9)
(2,10)	(2,5)	(2,6)	(2,7)	(2,8)	(2,9)
(3,10)	(3,11)	(3,6)	(3,7)	(3,8)	(3,9)
(4,10)	(4,11)	(4,12)	(4,7)	(4,8)	(4,9)
(5,10)	(5,11)	(5,12)	(1,10)	(5,8)	(5,9)
(6,10)	(6,11)	(6,12)	(3,12)	(2,11)	(6,9)

**FIGURE 2:**
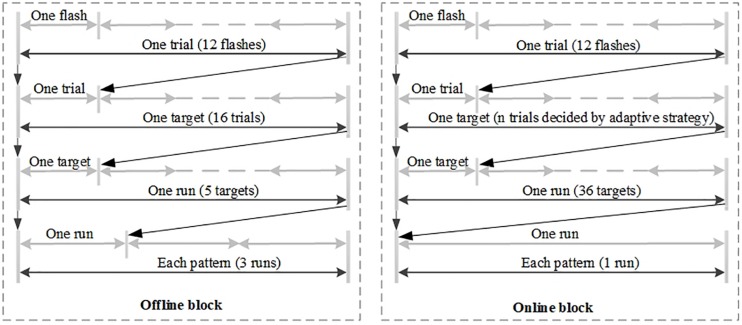
The flow diagram of the whole experiment.

The flow diagram of the experiment is shown in Figure 2. Each pattern was presented during both offline and online blocks. The offline block included three runs. In each run participants were asked to attempt to spell five targets without any break. After each offline run, participants had 3–5 min rest. Moreover, each target needed to be presented in 16 trials before it can be identified, and each trial consisted of 12 stimuli flashes. In the offline block, no feedback was displayed to the participants. The online block contained one run, which included a spelling task with 36 targets, each of which contained *n* trials, where *n* was decided by online adaptive strategy (Jin et al., 2011) for each target. Before each run began, the prompt box over the character indicated the target character.

Given the order that the three patterns were tested in could affect the performance, we kept split the participants into three, uniformly sized, groups. Each group was presented the three patterns in a different order. Table 2 lists the order of presentation of the three patterns for all 12 participants. Specifically, participants S1, S4, S5, and S8 attempted to use the RSF pattern, followed by the GSF pattern, and then the BSF pattern. Participants S2, S3, S6, and S11 used the GSF pattern, BSF pattern, and then the RSF pattern, Finally, participants S7, S9, S10, and S12 used the BSF pattern, RSF pattern, and then the GSF pattern (see Table 2).

**TABLE 2:** The order of patterns for 12 participants.

	**S1**	**S2**	**S3**	**S4**	**S5**	**S6**	**S7**	**S8**	**S9**	**S10**	**S11**	**S12**
RSF	1	3	3	1	1	3	2	1	2	2	3	2
GSF	2	1	1	2	2	1	3	2	3	3	1	3
BSF	3	2	2	3	3	2	1	3	1	1	2	1

*Note that “1” refers to the first pattern presented to the participant, and “2” refers to the second pattern and “3” denotes the last.*

### Stimulus Consistency

We prepared the interface composed of a black background and white characters, which was used to show a traditional P300 speller interface (Farwell and Donchin, 1988). In order to ensure the consistency of the color lightness and saturation across the three stimuli, we referred to G. Saravanan’s study (Saravanan et al., 2016) which transformed RGB values to the Hue, Saturation, and Luminance (HLS) color scale. The conversion formula is expressed in the following equation.

(1) $$R^{\prime }=R/255;G^{\prime }=G/255;B^{\prime }=B/255$$

(2) $$C_{m\,a\,x}=M\,A\,X\,\left(R^{\prime },G^{\prime },B^{\prime }\right)$$

(3) $$C_{m\,i\,n}=M\,I\,N\,\left(R^{\prime },G^{\prime },B^{\prime }\right)$$

(4) $$\Delta =C_{m\,a\,x}-C_{m\,i\,n}$$

The HSL values can be calculated by the following formula.

(5) $$H=\{\begin{array}{cc} 0^{{}^{\circ}}, & \Delta =0\\ 60^{{}^{\circ}}\times \left(\frac{G^{\prime }-B^{\prime }}{\Delta }+0\right), & C_{max}=R^{\prime }\\ 60^{{}^{\circ}}\times \left(\frac{B^{\prime }-R^{\prime }}{\Delta }+2\right), & C_{max}=G^{\prime }\\ 60^{{}^{\circ}}\times \left(\frac{R^{\prime }-G^{\prime }}{\Delta }+4\right), & C_{max}=B^{\prime } \end{array}$$

(6) $$S=\left\{ \begin{array}{cc} 0,\Delta =0\; & \\ \frac{\Delta }{1-\left|2\,L-1\right|},o\,t\,h\,e\,r\; & \end{array}\right.$$

(7) $$L=\left(C_{m\,a\,x}+C_{m\,i\,n}\right)/2$$

In this work, we calculated the corresponding values of hue, saturation, and luminance of the three stimuli. The three stimuli refer to the red (255,0,0), green (0,255,0), and blue (0,0,255) colors. It is noteworthy that the background of the interface was black with white characters and the three stimuli were consistent in saturation and luminance while differing in hue. This is shown in Table 3.

**TABLE 3:** The corresponding HSL value of the three stimuli.

	**R (red)**	**G (green)**	**B (blue)**	**H (hue)**	**S (saturation)**	**L (luminance)**
RSF	255	0	0	0	100	50
GSF	0	255	0	120	100	50
BSF	0	0	255	240	100	50

### Electroencephalogram Acquisition

**FIGURE 3:**
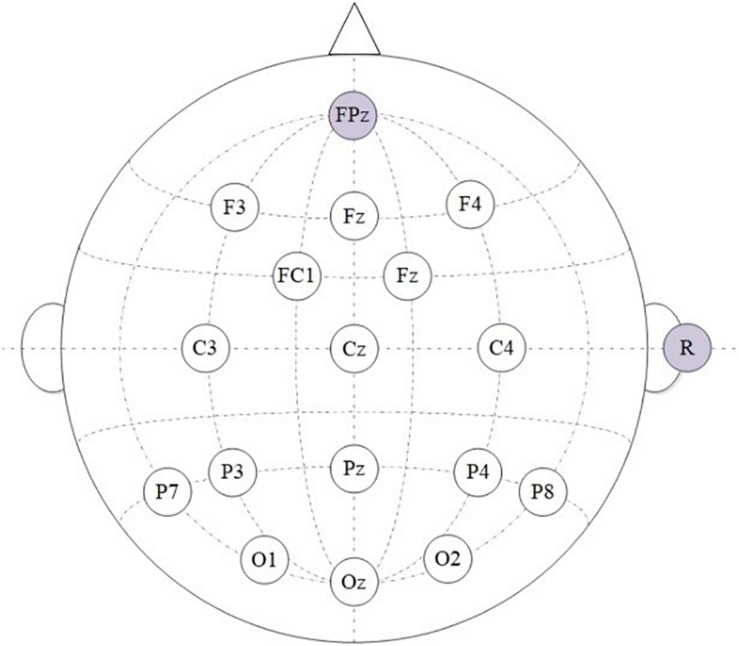
The configuration of the selected electrode positions from the 10–20 system.

These EEG signals were recorded with g.USBamp and g.EEGcap systems (Guger Technologies, Graz, Austria). The sample rate of the amplifier was set as 256 Hz, the sensitivity value was100μ*V*, and a third-order Butterworth band-pass filter was applied from 0.1 to 30 Hz (Munssinger et al., 2010; Halder et al., 2016). In this paper, we chose 16 electrode positions, based on the international 10–20 system (Jin et al., 2014a), which were positioned over areas of the brain associated with vision. These electrodes were Fz, F3, F4, FC1, FC2, C3, Cz, C4, P3, Pz, P4, P7, P8, O1, Oz, and O2. The ground electrode was placed at position FPz, while the reference electrode was placed on the right mastoid (R) (Jin et al., 2010, 2012, 2015). According to Petten and Kutas (1988), the use of the right mastoid reference leads to conclusions which are somewhat similar to those with the average of left and right mastoids. The electrode impedance was kept below 5 kΩ in the experiment (Munssinger et al., 2010). Figure 3 shows the configuration of the selected electrode positions.

### Feature Extraction and Classification

After completing the offline block, feature extraction is used to reduce dimensionality and hence computation time. Extracted features were used to construct the individual classifier model, which was applied during the online block. A band pass filter was applied to filter the EEG between 1 and 30 Hz to reduce high frequency noise. The filtering algorithm we applied was a third-order Butterworth filter. In order to eliminate the impact of electrical noise, the IIR notch filter of 50 Hz was also applied. In order to decrease dimensionality of the data and complexity of the classification model, the filtered EEG data was down-sampled from 256 to 36.6 Hz by taking every 7th sample.

The first 800 ms of EEG after stimulus presentation was extracted from each channel. This resulted in a feature vector of size 16 × 29, where 16 is the number of channels we used and 29 is the number of sample points recorded on each channel after down-sampling. Moreover, we used winsorizing to remove ocular artifacts by filtering amplitudes which were less than or greater than 10 and 90% of the amplitude distribution across the feature set (Jin et al., 2014b).

In this study, we applied Bayesian linear discriminant analysis (BLDA) to construct the individual classifier model which was used during the online block. Due to its regularization, it can avoid the problem of overfitting of high-dimensional data or noise interference. Hoffmann et al. (2008) first proposed BLDA and applied it to the P300-based BCI system effectively. In addition, after constructing the model, the score per flash was obtained. Within one trial, that is twelve flashes, the target flash should achieve the highest mark.

In accordance with widely used standardized metrics for assessing BCI performance, the classification accuracy and information transfer rate (ITR) are applied to assess the performance of our BCI. The ITR is defined as:

(8) $$B=l\,o\,g_{2}\,N+A\,c\,c*l\,o\,g_{2}\,A\,c\,c+\left(1-A\,c\,c\right)*l\,o\,g_{2}\,\frac{1-A\,c\,c}{N-1}$$

(9) $$I\,T\,R=B*\frac{60}{T}$$

where *N* represents the total number of targets, *Acc* denotes the classification accuracy, and *T* represents the time performing each trial.

### Online Adaptive System Setting

In order to improve system performance, an adaptive strategy was used with the online spelling system (Jin et al., 2011). In the online spelling system, the number of trials used to select each character is related to the classifier output after each trial. Specifically, when the classifier recognized the same character on two successive trials, no new flashes are needed and the recognized character is presented on the screen as feedback to the BCI user. If the number of trials needed to recognize a character reaches 16 without any pair of consecutive trials recognizing the same character, the classifier will automatically choose the target recognized in the final trial. For example, suppose that “A” is the target character which the classifier recognized in the first trial. If the character “A” was recognized again in the second trial, the final output will be “A”. We can describe this process via *cha*(*n*) = *cha*(*n*−1)[*cpsbreak*](1 < *n*≤16).

### Statistical Analysis

The One-Sample Ryan-Joiner test based on the correction of Shapiro-Wilk was used to analyze whether the samples were normally distributed. A Repeated-measures ANOVA (RM-ANOVA) was chosen to evaluate the effect of stimuli pattern. Mauchly’s test of sphericity was first used to check the data meets the assumptions of the RM-ANOVA. If the assumption was broken, Greenhouse-Geisser correction was performed to adjust the degrees of freedom. Finally, we applied Bonferroni multiple comparisons correction in *post hoc* tests (Kathner et al., 2015). The alpha level was set to 0.05 after Bonferroni correction.

## Results

### ERP Analysis

**FIGURE 4:**
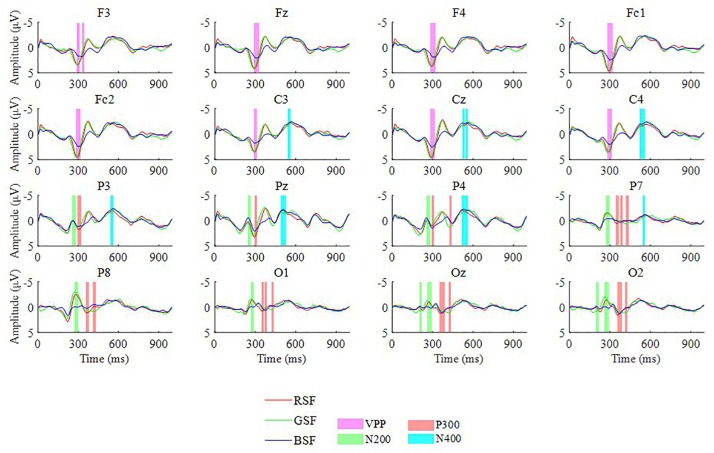
The grand averaged ERP amplitudes of targets for 10 participants over 16 electrodes among the three patterns.

Figure 4 illustrates the grand averaged ERP amplitudes in response to the target stimuli for ten participants over 16 electrodes across the three patterns, after applying baseline correction with a 100 ms pre-stimulus baseline. In Figure 4, the three colors of the curves in each channel illustrate the three different kinds of patterns respectively. Four color blocks lie around the peak point, which represents four types of potentials including the vertex positive potential (VPP), the N200, P300, and N400 potentials. We selected the latency of the potentials as the peak point with the range (min −10 ms, max + 10 ms). As we can see in Figure 4, the VPP components exist in frontal and central sites while the N200 and P300 components are centered over parietal and occipital areas. In addition, the peak amplitude of BSF curve performed lower than RSF and GSF curves (see Figure 4). According to studies of W. D. Wright (Gregory, 1973) and Fuortes (Fuortes et al., 1973), the human eye is composed of three color-sensitive cone-cell types (red, green, and blue). These three cone types have different responses for different stimulus wavelengths. Red cones are more sensitive to red color, green cones are more sensitive to green color. Among the three cone types, the red-cone presents the best response followed closely by the green-cone, with the blue cones having the lowest response, which may cause the difference. Furthermore, according to a RM-ANOVA, the P300 amplitude evoked by the RSF pattern is significantly larger than the other two patterns (*p* < 0.05) on parietal and occipital sites, corresponding to electrode P3, P7, Pz, P8, O1, Oz, and O_2_.

**FIGURE 5:**
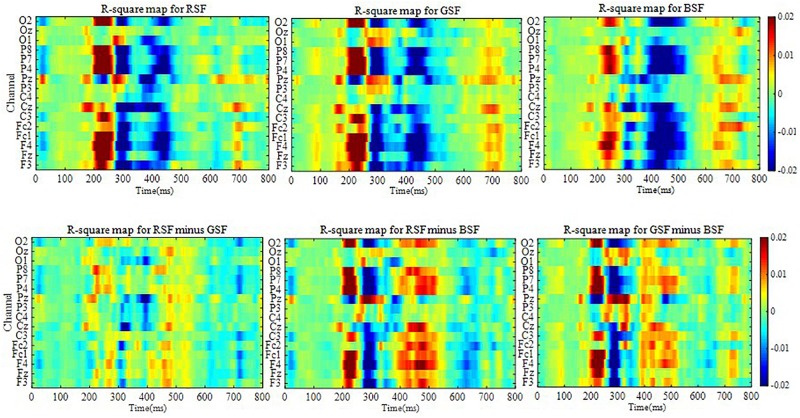
The signed R-squared value maps from 0 to 800 ms for 10 participants over 16 electrodes for each of the three patterns and for the differences of the three patterns.

Figure 5 shows the signed R-squared value maps from 0 to 800 ms for ten participants over 16 electrodes for each of the three patterns, which reflects the difference between the target and non-target stimuli over 16 channels. In order to show the difference among R-square map for RSF, GSF, and BSF patterns, the additional three R-square maps for the differences between RSF and GSF pattern, between RSF and BSF pattern and between GSF and BSF have also shown in Figure 5. The R-squared values of the ERPs evaluate the separation between target and non-target signals. The formula is given as:

(10) $$r^{2}={\left(\frac{\sqrt{N_{1}\,N_{2}}}{N_{1}+N_{2}}\cdot \frac{m\,e\,a\,n\,\left(X_{1}\right)-m\,e\,a\,n\,\left(X_{2}\right)}{s\,t\,d\,\left(X_{1}\,\cup X_{2}\right)}\right)}^{2}$$

where *X*_*1*_ and *X*_*2*_ refer to the features of class 1 and class 2 respectively, and *N*_*1*_ and *N*_*2*_ are the number of corresponding samples. In Figure 5, the darker the color, the more distinct the features.

### Classification Accuracy and Bit Rate

**FIGURE 6:**
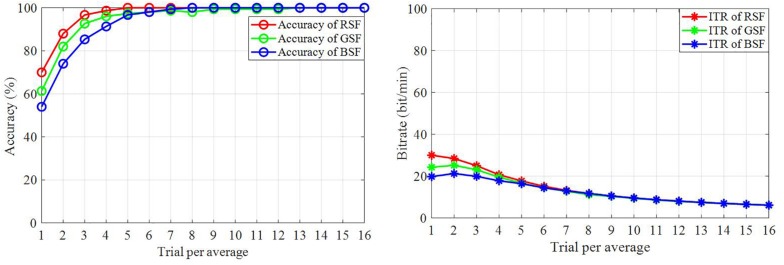
Classification accuracy and raw bit rate based on the offline data.

**FIGURE 7:**
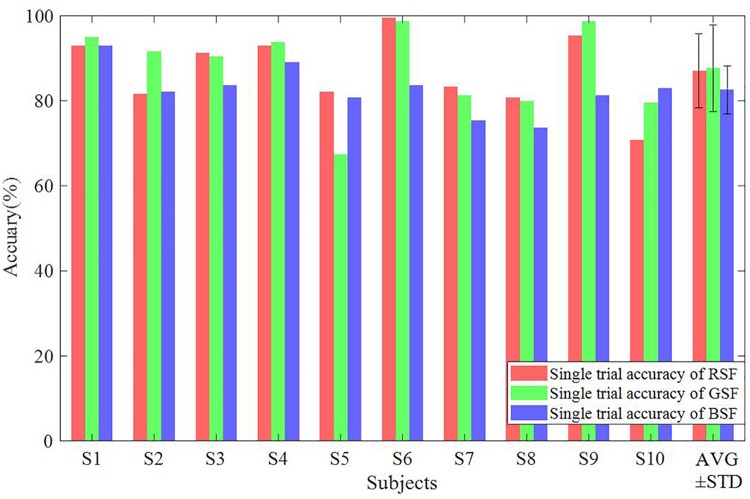
The classification accuracy based on offline single trial classification.

Figure 6 illustrates the classification accuracy and raw bit rates for each of the three patterns, which were overlapped and averaged from all trials for the ten participants based on the offline data. This valued were acquired from 15-fold cross-validation. As shown in Figure 6, the RSF pattern achieved the best offline accuracy and bit rate by averaging 16 trials. This pattern also used required the fewest the least trials to attain an accuracy of 100%. Figure 7 depicts the classification accuracy based on offline single trials, which shows no significant differences across the three patterns.

In order to observe the differences between the N200, VPP, P300, and N400 ERP components between the three patterns, we chose channel P8 for measuring the N200, Cz for measuring the VPP, Pz for measuring the P300 and Cz for measuring the N400 (Farwell and Donchin, 1988; Jeffreys and Tukmachi, 1992; Duncan et al., 2009). The selected channels generally cover the highest ERP amplitude of the corresponding component.

Table 4 describes the averaged amplitudes of the VPP on channel Cz, N200 on channel P8, P300 on channel Pz and N400 on channel Cz from the peak point ± 10 ms for the ten participants. The averaged values of VPP, P300, and N400 are largest when the RSF pattern is used, and the stability of the P300 during presentation of the RSF pattern is better than that the other patterns.

**TABLE 4:** The averaged amplitudes from each ERP peak point ± 10 ms of all participant.

		**RSF (μ*V*)**		**GSF (μ*V*)**		**BSF (μ*V*)**	
**Potential**	**Channel**	**Amplitude**	**STD**	**Amplitude**	**STD**	**Amplitude**	**STD**
VPP	Cz	5.40	1.99	5.38	2.31	3.35	1.49
N200	P8	–3.67	2.10	–4.25	1.90	–1.28	1.01
P300	Pz	3.46	0.99	3.12	1.47	2.84	1.02
N400	Cz	–4.33	1.53	–4.16	1.68	–3.97	1.52

**FIGURE 8:**
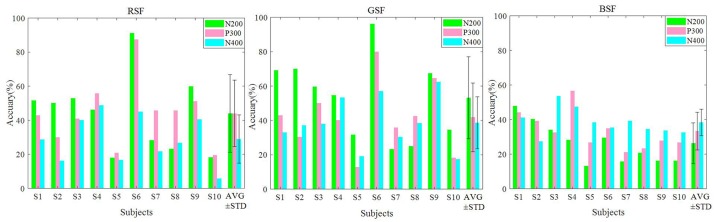
The contributions on N200, P300, and N400 time windows on the classification accuracy.

Figure 8 presents the averaged contributions of the N200, P300, and N400 components to the offline classification accuracy for the ten participants. The N200 had a latency of 150–300 ms after stimulation, the P300 had a latency of 300–450 ms, and the N400 had a latency of 350–600 ms (Zhou et al., 2016). The result of the three patterns all delineated N200 and P300 played a pivotal role in offline classification. Moreover, the N400 potential has positive effect on the offline classification accuracy.

### Online Analysis

**FIGURE 9:**
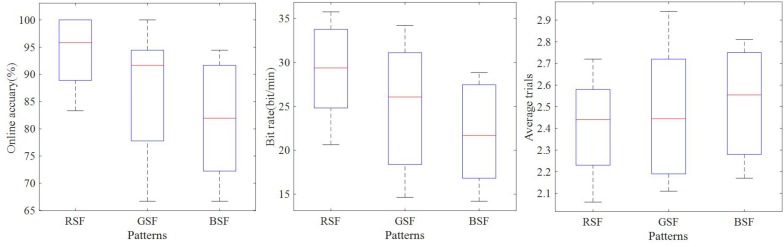
Boxplot of online classification accuracies, bit rates, and numbers of trials used to construct the averaged ERPs.

Table 5 shows the online accuracies, bit rates, and the averaged numbers of trials for participants S1–S10 for each of the three patterns. The calculated *p*-values indicate the significance of the difference between each pair of accuracies. Our one-way RM-ANOVA shows a significant effect of the factor “color” on the online accuracy (*F*(1.30,11.65) = 8.87,*p* < 0.05,*eta*^2^ = 0.50) and bit rate (*F*(1.11,10.02) = 9.25.*p* < 0.05,*eta*^2^ = 0.51). The online accuracy of the RSF pattern was significantly higher than that of the GSF pattern (*t* = 3.24,*p* < 0.05,*df* = 9) and the BSF pattern (*t* = 4.39,*p* < 0.05,*df* = 9). In addition, the bit rate of the RSF pattern was significantly higher than that of the GSF pattern (*t* = 5.77,*p* < 0.05,*df* = 9) and the BSF pattern (*t* = 3.93,*p* < 0.05,*df* = 9). However, there are no significant differences in the number of average trials needed for the classification across the three patterns. A boxplot of online accuracies is illustrated in Figure 9.

**TABLE 5:** Online accuracies, bit rates, and average trials analysis results.

	**Accuracy (%)**			**Bit rate (bit/min)**			**AVT**		
	**RSF**	**GSF**	**BSF**	**RSF**	**GSF**	**BSF**	**RSF**	**GSF**	**BSF**
S1	100.00	94.44	91.67	31.59	27.45	27.81	2.44	2.50	2.28
S2	97.22	86.11	88.89	29.60	24.66	26.32	2.44	2.31	2.28
S3	94.44	94.44	94.44	29.11	28.39	27.45	2.31	2.39	2.50
S4	100.00	94.44	91.67	33.75	31.09	28.83	2.23	2.11	2.17
S5	91.67	77.78	75.00	24.93	18.38	17.03	2.64	2.72	2.78
S6	100.00	97.22	86.11	35.74	32.26	25.40	2.06	2.17	2.22
S7	83.33	77.78	69.44	21.47	17.31	15.28	2.58	2.94	2.72
S8	88.89	88.89	72.22	24.80	22.49	16.81	2.47	2.81	2.61
S9	100.00	100.00	66.67	35.13	34.20	14.19	2.11	2.19	2.75
S10	83.33	66.67	77.78	20.63	14.64	17.93	2.72	2.64	2.81
AVG	**93.89**	87.78	81.39	**28.68**	25.09	21.70	**2.40**	2.48	2.51
STD	6.78	10.65	10.32	5.50	6.74	5.90	0.22	0.29	0.24
*p*	**RSF vs. GSF**	GSF vs. BSF	**RSF vs. BSF**	**RSF vs. GSF**	GSF vs. BSF	**RSF vs. BSF**	RSF vs. GSF	GSF vs. BSF	RSF vs. BSF
	**0.031**	0.392	**0**.**005**	**0.001**	0.417	**0.010**	0.535	1.000	0.464

*Note that AVT refers to the average number of trials used in online spelling. The *p*-value is obtained by applying Bonferroni correction. The bold values refer to the highest online accuracy, bit rate, and the smallest number of trials among three patterns. In the row of “value,” the bold item indicates which item is significant.*

### Participants’ Feedback

At the end of the whole experiment, every participant was asked to grade their perception of the tiredness and difficulty of each pattern. Tiredness and difficulty were each given a rating between 1 and 3. A score of 1 corresponded to a little, a score of 2 medium, and a score of 3 quite a lot of tiredness or difficulty. The questions were asked in Mandarin Chinese. For the sake of distinguishing the differences among three patterns, a non-parametric Friedman test was applied to reveal the differences in feedback. Table 6 delineates the feedback of all participants among three patterns. No significant difference (χ^2^ = 5.034,*p* > 0.05) was found between the patterns in terms of difficulty or tiredness (χ^2^=0.636,*p* > 0.05).

**TABLE 6:** The feedback of all participants for each of the three patterns.

	**S1**	**S2**	**S3**	**S4**	**S5**	**S6**	**S7**	**S8**	**S9**	**S10**	**AVG ± STD**
**Tiredness**											
RSF	2	1	1	1	2	2	3	2	1	2	1.7 ± 0.67
GSF	1	2	1	2	1	1	2	1	1	2	1.4 ± 0.52
BSF	2	2	2	1	3	2	2	3	1	3	2.1 ± 0.74
**Difficulty**											
RSF	1	1	1	2	2	2	2	2	1	1	1.5 ± 0.53
GSF	1	1	1	1	1	2	3	1	2	2	1.5 ± 0.71
BSF	1	2	1	1	3	2	2	2	1	2	1.7 ± 0.67

*Note that a score of “1” denotes a few, “2” medium, and “3” many. AVG denotes the average and STD denotes the standard deviation.*

## Discussion

ERP-based BCI systems have been widely investigated over many years and some researchers have designed novel stimulus paradigms to optimize system performance. Previous work has indicated that familiar faces, colored green, may be used as a part of the ERP-based BCI display pattern to achieve higher performance than other display patterns, such as the familiar face pattern based in P300-speller BCI system (Li et al., 2015). Therefore, we evaluated how this paradigm was influenced by other colors (red, green, and blue).

Related studies have indicated that, when familiar faces are used as stimuli, they may strongly elicit several ERPs, including the VPP, N200, P300, and N400 components. Cheng et al. (2017) reported that the semitransparent face pattern can evoke larger N200 components, which can contribute to improving classification accuracy. Eimer (2000) revealed that familiar faces could elicit an N400 in parietal and central cortical areas. In addition, the VPP component remarkably increase for face-related stimuli over frontal and central sites (Zhang et al., 2012). Among the three patterns evaluated in this study, we found all the ERP components, shown in Figure 4. Moreover, we can see from Figure 8 that the P300, N200, and the N400 all contribute to the classification accuracy. The results also indicate that the RSF pattern could elicit larger P300 potentials on parietal and occipital areas.

Generally, the performance of a BCI can be evaluated by online accuracy and ITR. The results listed in Table 5 indicate that the RSF pattern achieved the highest online averaged accuracy of 93.89%, followed by the GSF pattern with 87.78%, while the lowest accuracy was achieved with the BSF pattern (81.39%). Four of the participants using the RSF pattern obtained 100% online accuracy. Furthermore, the online accuracy achieved with the RSF is significantly higher than that achieved with the GSF pattern (*p* < 0.05) and the BSF pattern (*p* < 0.05). In addition, significant differences in bit rate were found between the RSF and GSF patterns (*p* < 0.05) and between RSF and BSF patterns (*p* < 0.05). The averaged bit rate of the RSF pattern was 38.45 bit/min, and the bit rate of the GSF pattern was 33.71 bit/min, while the bit rate of the BSF was 28.76 bit/min. Due to the averaged presentation order of the three patterns for all participants, the effect caused by the order of pattern presentation can be ignored. Consequently, we may conclude that the RSF pattern yielded the best performance of the three patterns.

In order to further explain the findings, it is necessary to consider relevant psychological and physiological studies. Research has shown that long-wavelength colors (e.g., red and yellow) are more arousing than short-wavelength colors (e.g., blue and green) (Wilson, 1966). In our experiment, each face stimulus was presented for more than half an hour, which may induce some effects on the emotions of the participants. Additionally, an association has been reported between colors and physiological indices of cognition. For instance, the color red is frequently associated with fire and blood which can lead to excitement and fear (Kaiser, 1984; Camgöz et al., 2004). Sorokowski and Szmajke (2011) found that red could improve performance in a target-hitting task. This result indicated that participants attempting to hit a red moving objects can achieve better performance than participants attempting to hit blue or black targets.

In previous studies, the green/blue chromatic flicker as a visual stimulus yielded an 80.6% online accuracy (Takano et al., 2009). Li et al. (2015) proposed that a translucent green familiar face spelling paradigm could achieve an 86.1% averaged online accuracy. This SSVEP-based BCI system used LEDs of four different colors (red, green, blue, and yellow) flickering at four distinct frequencies (8, 11, 13, and 15 Hz) (Mouli et al., 2013). It was observed that the red color obtained the highest accuracy and bit rate in most frequencies. Therefore, a novel spelling pattern that combines chromatic difference (RGB) with semitransparent faces resulted in consistency and efficiency in online BCI performance and offline ERP waveform detection.

## Conclusion

In the present work, we combined chromatic difference (RGB) with semitransparent face stimuli to explore the performance of different colored stimuli patterns in an ERP based BCI system. The results demonstrated that the RSF pattern yielded the best averaged online accuracy and ITR. In future work, we will attempt to train offline models using neural networks to boost the classification performance. In addition, according to Xu’s study (Xu et al., 2018), a new BCI speller based on miniature asymmetric visual evoked potentials (aVEPs) could reduce visual fatigue for users. This demonstrates the feasibility to implement an efficient BCI system. We will further explore the effect of color preference on system performance and take user-friendliness into account to improve the usability of BCI systems. This may have a clinically significant impact by increasing communication speed and accuracy of the P300-speller for patients with severe motor impairment.

## Data Availability Statement

The datasets generated for this study are available on request to the corresponding author.

## Ethics Statement

The studies involving human participants were reviewed and approved by the Ethics Committee of East China University of Science and Technology. The patients/participants provided their written informed consent to participate in this study. Written informed consent was obtained from the individual(s) for the publication of any potentially identifiable images or data included in this article.

## Author Contributions

SL was the main author to raise the idea of the manuscript, design the whole experiment, and collect the original dataset. All authors contributed to the manuscript revision, read, and approved the submitted version.

## Conflict of Interest

The authors declare that the research was conducted in the absence of any commercial or financial relationships that could be construed as a potential conflict of interest.
